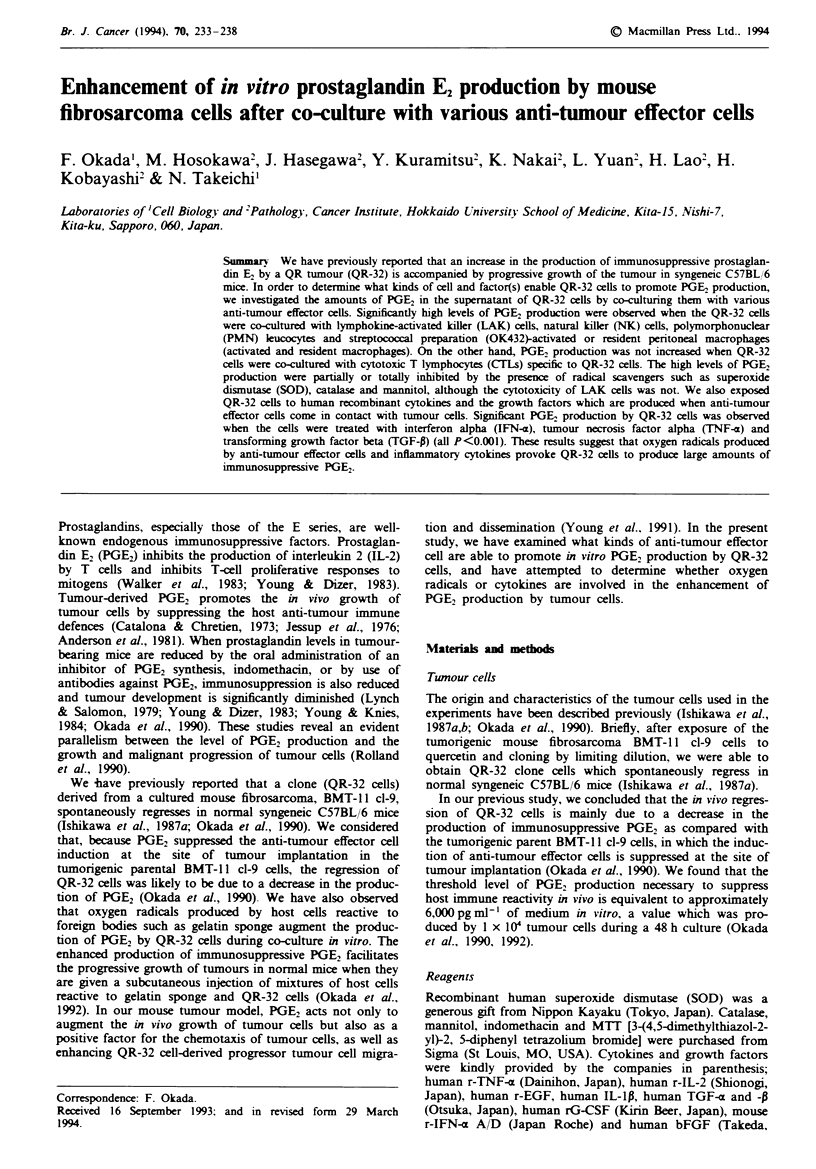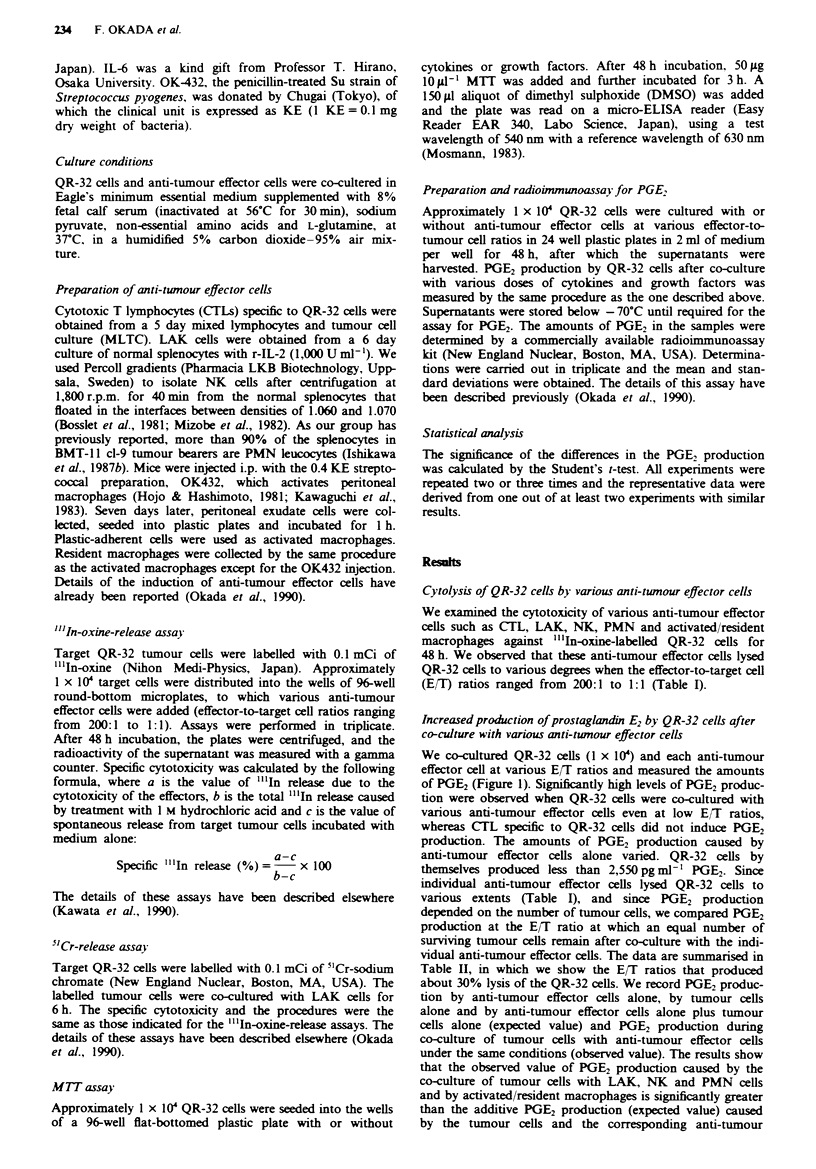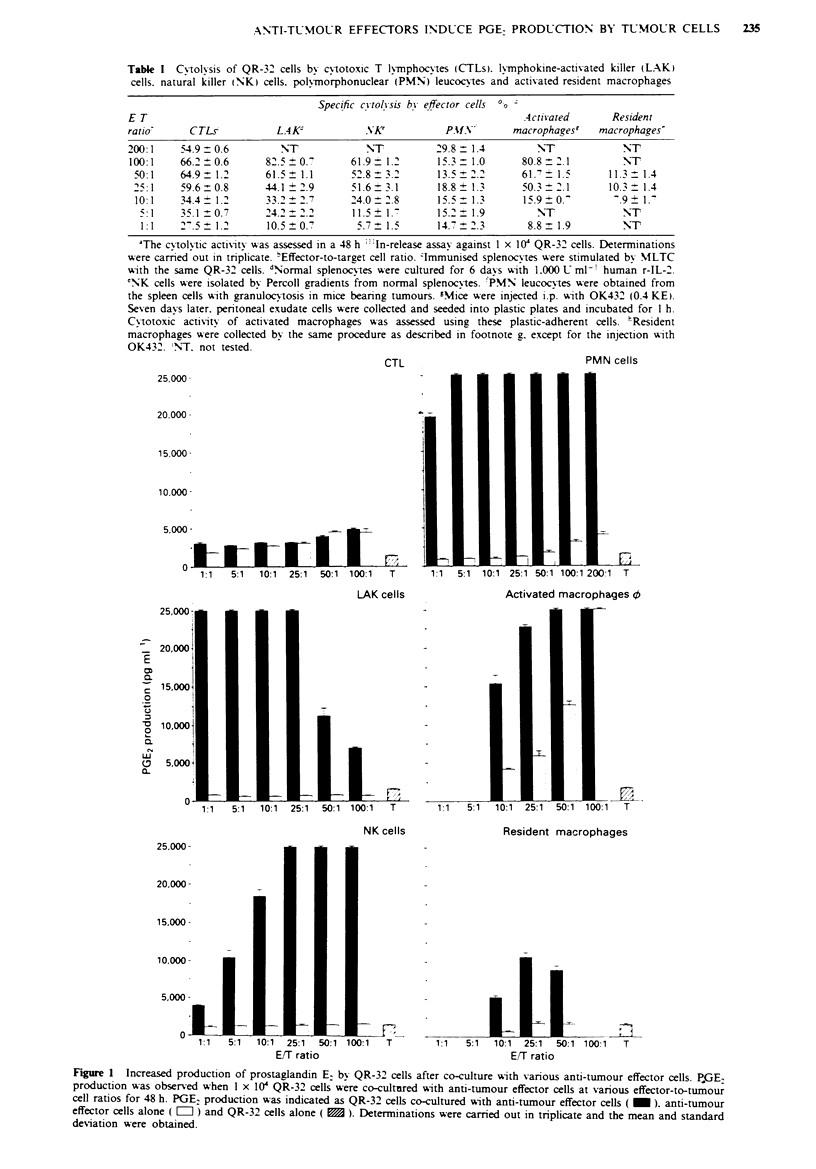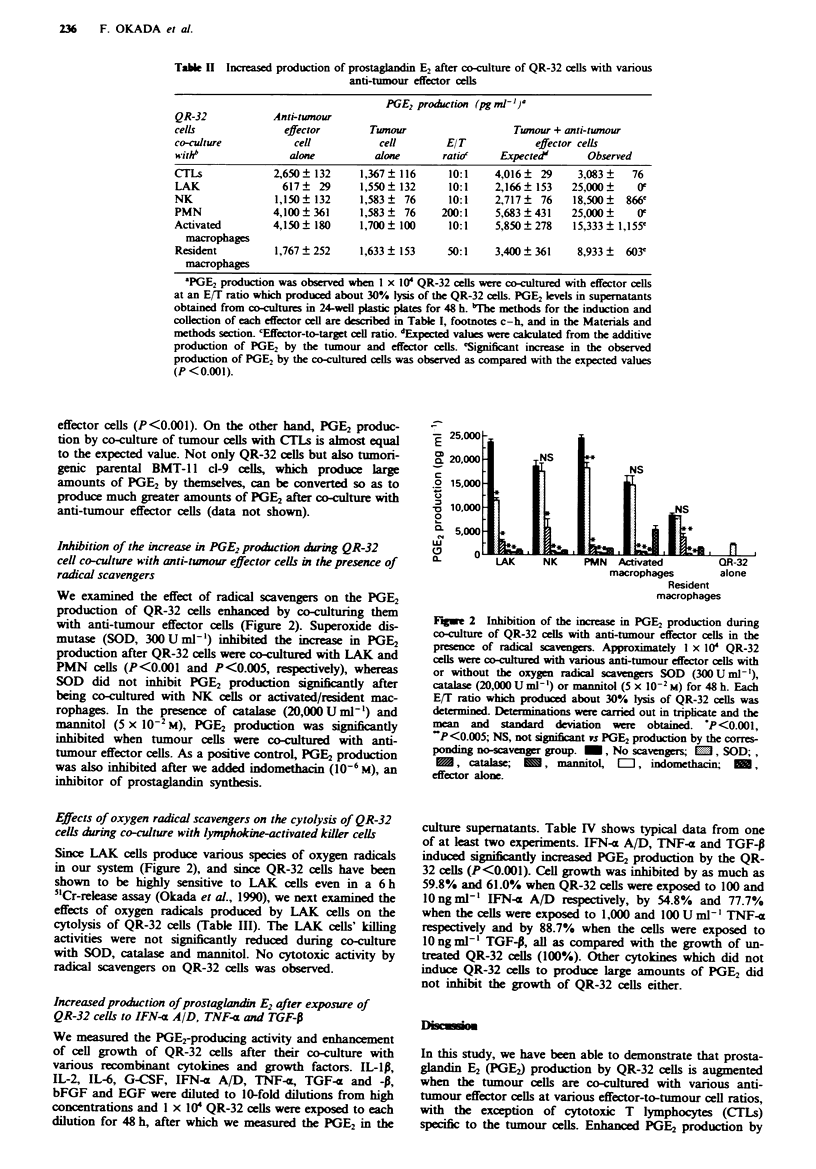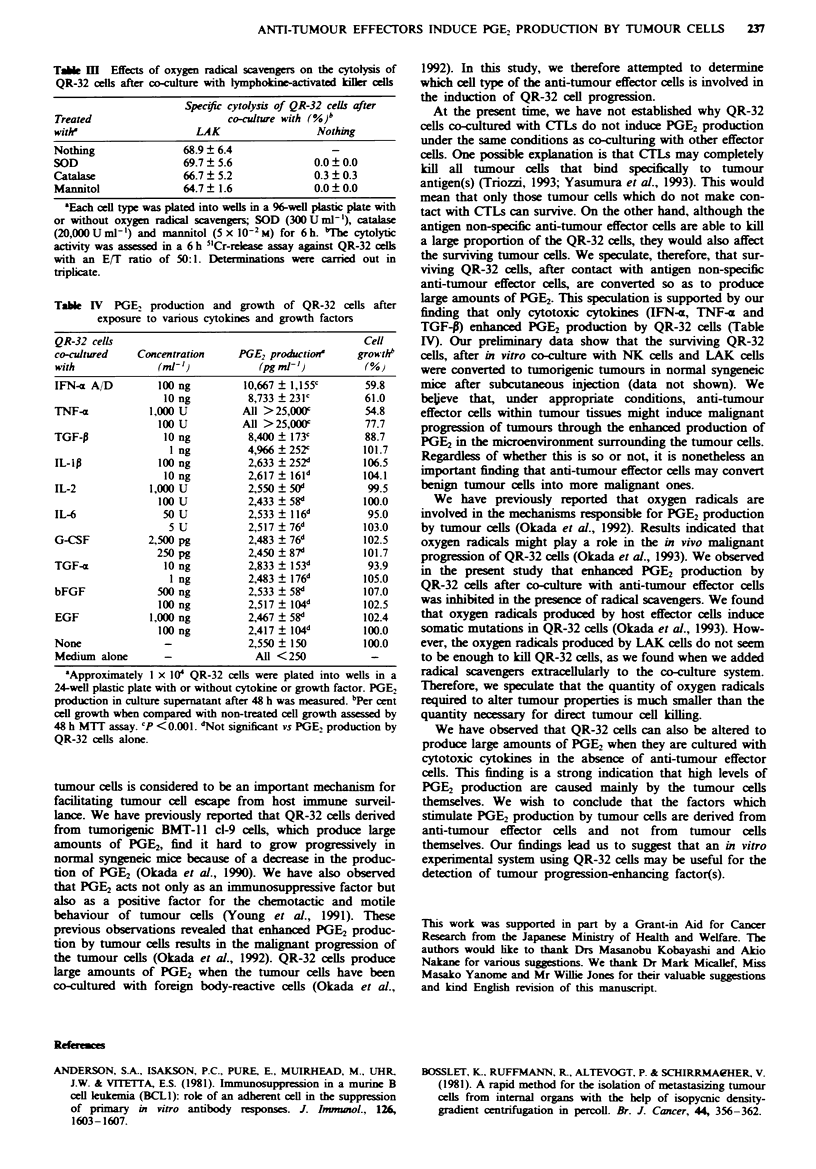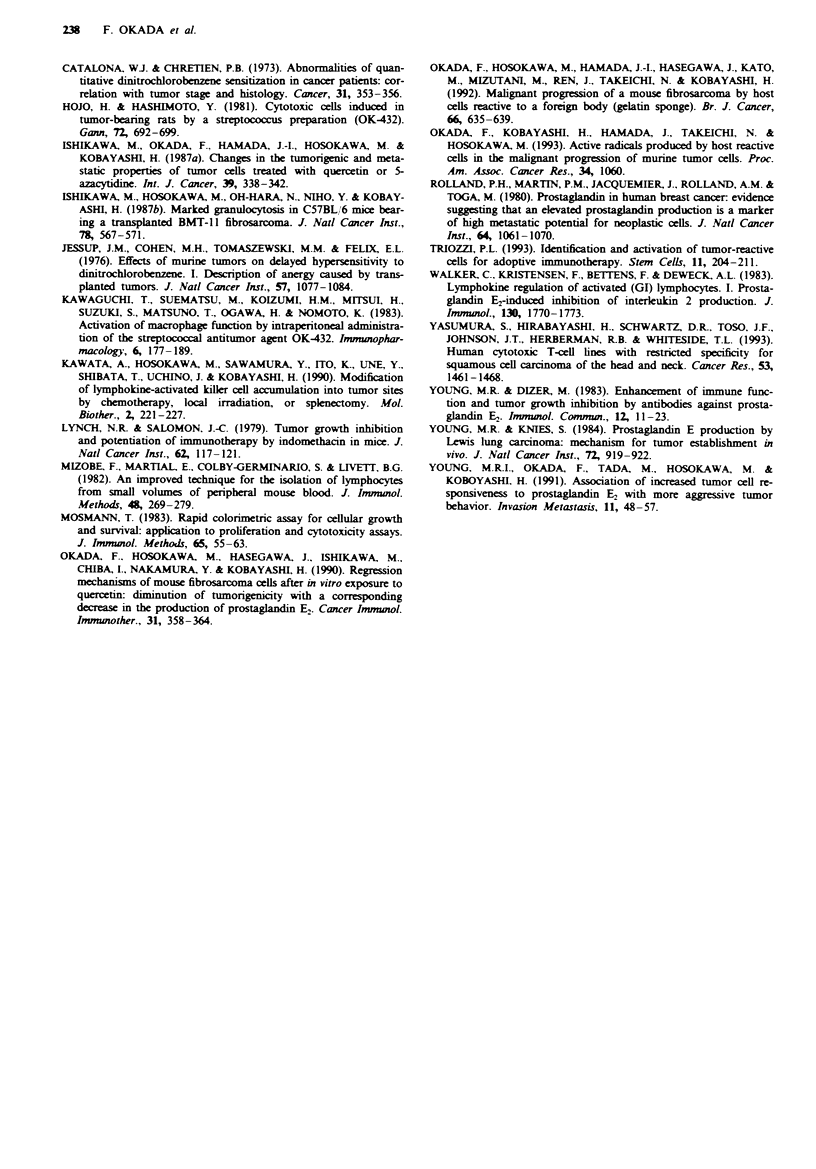# Enhancement of in vitro prostaglandin E2 production by mouse fibrosarcoma cells after co-culture with various anti-tumour effector cells.

**DOI:** 10.1038/bjc.1994.285

**Published:** 1994-08

**Authors:** F. Okada, M. Hosokawa, J. Hasegawa, Y. Kuramitsu, K. Nakai, L. Yuan, H. Lao, H. Kobayashi, N. Takeichi

**Affiliations:** Laboratory of Cell Biology, Hokkaido University School of Medicine, Sapporo, Japan.

## Abstract

We have previously reported that an increase in the production of immunosuppressive prostaglandin E2 by a QR tumour (QR-32) is accompanied by progressive growth of the tumour in syngeneic C57BL/6 mice. In order to determine what kinds of cell and factor(s) enable QR-32 cells to promote PGE2 production, we investigated the amounts of PGE2 in the supernatant of QR-32 cells by co-culturing them with various anti-tumour effector cells. Significantly high levels of PGE2 production were observed when the QR-32 cells were co-cultured with lymphokine-activated killer (LAK) cells, natural killer (NK) cells, polymorphonuclear (PMN) leucocytes and streptococcal preparation (OK432)-activated or resident peritoneal macrophages (activated and resident macrophages). On the other hand, PGE2 production was not increased when QR-32 cells were co-cultured with cytotoxic T lymphocytes (CTLs) specific to QR-32 cells. The high levels of PGE2 production were partially or totally inhibited by the presence of radical scavengers such as superoxide dismutase (SOD), catalase and mannitol, although the cytotoxicity of LAK cells was not. We also exposed QR-32 cells to human recombinant cytokines and the growth factors which are produced when anti-tumour effector cells come in contact with tumour cells. Significant PGE2 production by QR-32 cells was observed when the cells were treated with interferon alpha (IFN-alpha), tumour necrosis factor alpha (TNF-alpha) and transforming growth factor beta (TGF-beta) (all P < 0.001). These results suggest that oxygen radicals produced by anti-tumour effector cells and inflammatory cytokines provoke QR-32 cells to produce large amounts of immunosuppressive PGE2.


Br. J. Cancer (1994), 70, 233-238 © Macmillan Press Ltd., 1994

### Enhancement of *in vitro* prostaglandin $E_2$ production by mouse fibrosarcoma cells after co-culture with various anti-tumour effector cells

F. Okada[1], M. Hosokawa[2], J. Hasegawa[2], Y. Kuramitsu[2], K. Nakai[2], L. Yuan[2], H. Lao[2], H. Kobayashi[2] & N. Takeichi[1]

*Laboratories of [1]Cell Biology and [2]Pathology, Cancer Institute, Hokkaido University School of Medicine, Kita-15, Nishi-7, Kita-ku, Sapporo, 060, Japan.*

**Summary** We have previously reported that an increase in the production of immunosuppressive prostaglandin $E_2$ by a QR tumour (QR-32) is accompanied by progressive growth of the tumour in syngeneic C57BL/6 mice. In order to determine what kinds of cell and factor(s) enable QR-32 cells to promote $PGE_2$ production, we investigated the amounts of $PGE_2$ in the supernatant of QR-32 cells by co-culturing them with various anti-tumour effector cells. Significantly high levels of $PGE_2$ production were observed when the QR-32 cells were co-cultured with lymphokine-activated killer (LAK) cells, natural killer (NK) cells, polymorphonuclear (PMN) leucocytes and streptococcal preparation (OK432)-activated or resident peritoneal macrophages (activated and resident macrophages). On the other hand, $PGE_2$ production was not increased when QR-32 cells were co-cultured with cytotoxic T lymphocytes (CTLs) specific to QR-32 cells. The high levels of $PGE_2$ production were partially or totally inhibited by the presence of radical scavengers such as superoxide dismutase (SOD), catalase and mannitol, although the cytotoxicity of LAK cells was not. We also exposed QR-32 cells to human recombinant cytokines and the growth factors which are produced when anti-tumour effector cells come in contact with tumour cells. Significant $PGE_2$ production by QR-32 cells was observed when the cells were treated with interferon alpha (IFN-α), tumour necrosis factor alpha (TNF-α) and transforming growth factor beta (TGF-β) (all $P<0.001$). These results suggest that oxygen radicals produced by anti-tumour effector cells and inflammatory cytokines provoke QR-32 cells to produce large amounts of immunosuppressive $PGE_2$.

Prostaglandins, especially those of the E series, are well-known endogenous immunosuppressive factors. Prostaglandin $E_2$ ($PGE_2$) inhibits the production of interleukin 2 (IL-2) by T cells and inhibits T-cell proliferative responses to mitogens (Walker *et al.*, 1983; Young & Dizer, 1983). Tumour-derived $PGE_2$ promotes the *in vivo* growth of tumour cells by suppressing the host anti-tumour immune defences (Catalona & Chretien, 1973; Jessup *et al.*, 1976; Anderson *et al.*, 1981). When prostaglandin levels in tumour-bearing mice are reduced by the oral administration of an inhibitor of $PGE_2$ synthesis, indomethacin, or by use of antibodies against $PGE_2$, immunosuppression is also reduced and tumour development is significantly diminished (Lynch & Salomon, 1979; Young & Dizer, 1983; Young & Knies, 1984; Okada *et al.*, 1990). These studies reveal an evident parallelism between the level of $PGE_2$ production and the growth and malignant progression of tumour cells (Rolland *et al.*, 1990).

We have previously reported that a clone (QR-32 cells) derived from a cultured mouse fibrosarcoma, BMT-11 cl-9, spontaneously regresses in normal syngeneic C57BL/6 mice (Ishikawa *et al.*, 1987*a*; Okada *et al.*, 1990). We considered that, because $PGE_2$ suppressed the anti-tumour effector cell induction at the site of tumour implantation in the tumorigenic parental BMT-11 cl-9 cells, the regression of QR-32 cells was likely to be due to a decrease in the production of $PGE_2$ (Okada *et al.*, 1990). We have also observed that oxygen radicals produced by host cells reactive to foreign bodies such as gelatin sponge augment the production of $PGE_2$ by QR-32 cells during co-culture *in vitro*. The enhanced production of immunosuppressive $PGE_2$ facilitates the progressive growth of tumours in normal mice when they are given a subcutaneous injection of mixtures of host cells reactive to gelatin sponge and QR-32 cells (Okada *et al.*, 1992). In our mouse tumour model, $PGE_2$ acts not only to augment the *in vivo* growth of tumour cells but also as a positive factor for the chemotaxis of tumour cells, as well as enhancing QR-32 cell-derived progressor tumour cell migration and dissemination (Young *et al.*, 1991). In the present study, we have examined what kinds of anti-tumour effector cell are able to promote *in vitro* $PGE_2$ production by QR-32 cells, and have attempted to determine whether oxygen radicals or cytokines are involved in the enhancement of $PGE_2$ production by tumour cells.

### Materials and methods

### Tumour cells

The origin and characteristics of the tumour cells used in the experiments have been described previously (Ishikawa *et al.*, 1987*a*,*b*; Okada *et al.*, 1990). Briefly, after exposure of the tumorigenic mouse fibrosarcoma BMT-11 cl-9 cells to quercetin and cloning by limiting dilution, we were able to obtain QR-32 clone cells which spontaneously regress in normal syngeneic C57BL/6 mice (Ishikawa *et al.*, 1987*a*).

In our previous study, we concluded that the *in vivo* regression of QR-32 cells is mainly due to a decrease in the production of immunosuppressive $PGE_2$ as compared with the tumorigenic parent BMT-11 cl-9 cells, in which the induction of anti-tumour effector cells is suppressed at the site of tumour implantation (Okada *et al.*, 1990). We found that the threshold level of $PGE_2$ production necessary to suppress host immune reactivity *in vivo* is equivalent to approximately 6,000 pg $ml^{-1}$ of medium *in vitro*, a value which was produced by $1 \times 10^4$ tumour cells during a 48 h culture (Okada *et al.*, 1990, 1992).

### Reagents

Recombinant human superoxide dismutase (SOD) was a generous gift from Nippon Kayaku (Tokyo, Japan). Catalase, mannitol, indomethacin and MTT [3-(4,5-dimethylthiazol-2-yl)-2, 5-diphenyl tetrazolium bromide] were purchased from Sigma (St Louis, MO, USA). Cytokines and growth factors were kindly provided by the companies in parenthesis; human r-TNF-α (Dainihon, Japan), human r-IL-2 (Shionogi, Japan), human r-EGF, human IL-1β, human TGF-α and -β (Otsuka, Japan), human rG-CSF (Kirin Beer, Japan), mouse r-IFN-α A/D (Japan Roche) and human bFGF (Takeda,

Correspondence: F. Okada.
Received 16 September 1993; and in revised form 29 March 1994.

Japan). IL-6 was a kind gift from Professor T. Hirano, Osaka University. OK-432, the penicillin-treated Su strain of *Streptococcus pyogenes*, was donated by Chugai (Tokyo), of which the clinical unit is expressed as KE (1 KE = 0.1 mg dry weight of bacteria).

### Culture conditions

QR-32 cells and anti-tumour effector cells were co-cultered in Eagle's minimum essential medium supplemented with 8% fetal calf serum (inactivated at 56°C for 30 min), sodium pyruvate, non-essential amino acids and L-glutamine, at 37°C, in a humidified 5% carbon dioxide-95% air mixture.

### Preparation of anti-tumour effector cells

Cytotoxic T lymphocytes (CTLs) specific to QR-32 cells were obtained from a 5 day mixed lymphocytes and tumour cell culture (MLTC). LAK cells were obtained from a 6 day culture of normal splenocytes with r-IL-2 (1,000 U $ml^{-1}$). We used Percoll gradients (Pharmacia LKB Biotechnology, Uppsala, Sweden) to isolate NK cells after centrifugation at 1,800 r.p.m. for 40 min from the normal splenocytes that floated in the interfaces between densities of 1.060 and 1.070 (Bosslet *et al.*, 1981; Mizobe *et al.*, 1982). As our group has previously reported, more than 90% of the splenocytes in BMT-11 cl-9 tumour bearers are PMN leucocytes (Ishikawa *et al.*, 1987*b*). Mice were injected i.p. with the 0.4 KE streptococcal preparation, OK432, which activates peritoneal macrophages (Hojo & Hashimoto, 1981; Kawaguchi *et al.*, 1983). Seven days later, peritoneal exudate cells were collected, seeded into plastic plates and incubated for 1 h. Plastic-adherent cells were used as activated macrophages. Resident macrophages were collected by the same procedure as the activated macrophages except for the OK432 injection. Details of the induction of anti-tumour effector cells have already been reported (Okada *et al.*, 1990).

### $^{111}$In-oxine-release assay

Target QR-32 tumour cells were labelled with 0.1 mCi of $^{111}$In-oxine (Nihon Medi-Physics, Japan). Approximately $1 \times 10^4$ target cells were distributed into the wells of 96-well round-bottom microplates, to which various anti-tumour effector cells were added (effector-to-target cell ratios ranging from 200:1 to 1:1). Assays were performed in triplicate. After 48 h incubation, the plates were centrifuged, and the radioactivity of the supernatant was measured with a gamma counter. Specific cytotoxicity was calculated by the following formula, where $a$ is the value of $^{111}$In release due to the cytotoxicity of the effectors, $b$ is the total $^{111}$In release caused by treatment with 1 M hydrochloric acid and $c$ is the value of spontaneous release from target tumour cells incubated with medium alone:

$$\text{Specific }^{111}\text{In release (\%)} = \frac{a-c}{b-c} \times 100$$

The details of these assays have been described elsewhere (Kawata *et al.*, 1990).

### $^{51}$Cr-release assay

Target QR-32 cells were labelled with 0.1 mCi of $^{51}$Cr-sodium chromate (New England Nuclear, Boston, MA, USA). The labelled tumour cells were co-cultured with LAK cells for 6 h. The specific cytotoxicity and the procedures were the same as those indicated for the $^{111}$In-oxine-release assays. The details of these assays have been described elsewhere (Okada *et al.*, 1990).

### MTT assay

Approximately $1 \times 10^4$ QR-32 cells were seeded into the wells of a 96-well flat-bottomed plastic plate with or without cytokines or growth factors. After 48 h incubation, 50 µg 10 $\mu l^{-1}$ MTT was added and further incubated for 3 h. A 150 µl aliquot of dimethyl sulphoxide (DMSO) was added and the plate was read on a micro-ELISA reader (Easy Reader EAR 340, Labo Science, Japan), using a test wavelength of 540 nm with a reference wavelength of 630 nm (Mosmann, 1983).

### Preparation and radioimmunoassay for $PGE_2$

Approximately $1 \times 10^4$ QR-32 cells were cultured with or without anti-tumour effector cells at various effector-to-tumour cell ratios in 24 well plastic plates in 2 ml of medium per well for 48 h, after which the supernatants were harvested. $PGE_2$ production by QR-32 cells after co-culture with various doses of cytokines and growth factors was measured by the same procedure as the one described above. Supernatants were stored below −70°C until required for the assay for $PGE_2$. The amounts of $PGE_2$ in the samples were determined by a commercially available radioimmunoassay kit (New England Nuclear, Boston, MA, USA). Determinations were carried out in triplicate and the mean and standard deviations were obtained. The details of this assay have been described previously (Okada *et al.*, 1990).

### Statistical analysis

The significance of the differences in the $PGE_2$ production was calculated by the Student's $t$-test. All experiments were repeated two or three times and the representative data were derived from one out of at least two experiments with similar results.

### Results

### Cytolysis of QR-32 cells by various anti-tumour effector cells

We examined the cytotoxicity of various anti-tumour effector cells such as CTL, LAK, NK, PMN and activated/resident macrophages against $^{111}$In-oxine-labelled QR-32 cells for 48 h. We observed that these anti-tumour effector cells lysed QR-32 cells to various degrees when the effector-to-target cell (E/T) ratios ranged from 200:1 to 1:1 (Table I).

### Increased production of prostaglandin $E_2$ by QR-32 cells after co-culture with various anti-tumour effector cells

We co-cultured QR-32 cells ($1 \times 10^4$) and each anti-tumour effector cell at various E/T ratios and measured the amounts of $PGE_2$ (Figure 1). Significantly high levels of $PGE_2$ production were observed when QR-32 cells were co-cultured with various anti-tumour effector cells even at low E/T ratios, whereas CTL specific to QR-32 cells did not induce $PGE_2$ production. The amounts of $PGE_2$ production caused by anti-tumour effector cells alone varied. QR-32 cells by themselves produced less than 2,550 pg $ml^{-1}$ $PGE_2$. Since individual anti-tumour effector cells lysed QR-32 cells to various extents (Table I), and since $PGE_2$ production depended on the number of tumour cells, we compared $PGE_2$ production at the E/T ratio at which an equal number of surviving tumour cells remain after co-culture with the individual anti-tumour effector cells. The data are summarised in Table II, in which we show the E/T ratios that produced about 30% lysis of the QR-32 cells. We record $PGE_2$ production by anti-tumour effector cells alone, by tumour cells alone and by anti-tumour effector cells alone plus tumour cells alone (expected value) and $PGE_2$ production during co-culture of tumour cells with anti-tumour effector cells under the same conditions (observed value). The results show that the observed value of $PGE_2$ production caused by the co-culture of tumour cells with LAK, NK and PMN cells and by activated/resident macrophages is significantly greater than the additive $PGE_2$ production (expected value) caused by the tumour cells and the corresponding anti-tumour

**Table I** Cytolysis of QR-32 cells by cytotoxic T lymphocytes (CTLs), lymphokine-activated killer (LAK) cells, natural killer (NK) cells, polymorphonuclear (PMN) leucocytes and activated resident macrophages

| | Specific cytolysis by effector cells (%)[a] | | | | | |
|---|---|---|---|---|---|---|
| *E/T ratio*[b] | *CTLs*[c] | *LAK*[d] | *NK*[e] | *PMN*[f] | *Activated macrophages*[g] | *Resident macrophages*[h] |
| 200:1 | 54.9 ± 0.6 | NT[i] | NT | 29.8 ± 1.4 | NT | NT |
| 100:1 | 66.2 ± 0.6 | 82.5 ± 0.7 | 61.9 ± 1.2 | 15.3 ± 1.0 | 80.8 ± 2.1 | NT |
| 50:1 | 64.9 ± 1.2 | 61.5 ± 1.1 | 52.8 ± 3.2 | 13.5 ± 2.2 | 61.7 ± 1.5 | 11.3 ± 1.4 |
| 25:1 | 59.6 ± 0.8 | 44.1 ± 2.9 | 51.6 ± 3.1 | 18.8 ± 1.3 | 50.3 ± 2.1 | 10.3 ± 1.4 |
| 10:1 | 34.4 ± 1.2 | 33.2 ± 2.7 | 24.0 ± 2.8 | 15.5 ± 1.3 | 15.9 ± 0.7 | 7.9 ± 1.7 |
| 5:1 | 35.1 ± 0.7 | 24.2 ± 2.2 | 11.5 ± 1.7 | 15.2 ± 1.9 | NT | NT |
| 1:1 | 27.5 ± 1.2 | 10.5 ± 0.7 | 5.7 ± 1.5 | 14.7 ± 2.3 | 8.8 ± 1.9 | NT |

[a]The cytolytic activity was assessed in a 48 h $^{111}$In-release assay against $1 \times 10^4$ QR-32 cells. Determinations were carried out in triplicate. [b]Effector-to-target cell ratio. [c]Immunised splenocytes were stimulated by MLTC with the same QR-32 cells. [d]Normal splenocytes were cultured for 6 days with 1,000 U ml$^{-1}$ human r-IL-2. [e]NK cells were isolated by Percoll gradients from normal splenocytes. [f]PMN leucocytes were obtained from the spleen cells with granulocytosis in mice bearing tumours. [g]Mice were injected i.p. with OK432 (0.4 KE). Seven days later, peritoneal exudate cells were collected and seeded into plastic plates and incubated for 1 h. Cytotoxic activity of activated macrophages was assessed using these plastic-adherent cells. [h]Resident macrophages were collected by the same procedure as described in footnote g, except for the injection with OK432. [i]NT, not tested.

**Figure 1** Increased production of prostaglandin E$_2$ by QR-32 cells after co-culture with various anti-tumour effector cells. PGE$_2$ production was observed when $1 \times 10^4$ QR-32 cells were co-cultured with anti-tumour effector cells at various effector-to-tumour cell ratios for 48 h. PGE$_2$ production was indicated as QR-32 cells co-cultured with anti-tumour effector cells ( ■ ), anti-tumour effector cells alone ( □ ) and QR-32 cells alone ( ▨ ). Determinations were carried out in triplicate and the mean and standard deviation were obtained.

**Table II** Increased production of prostaglandin $E_2$ after co-culture of QR-32 cells with various anti-tumour effector cells

| | $PGE_2$ production ($pg\ ml^{-1}$)[a] | | | | |
|---|---|---|---|---|---|
| *QR-32 cells co-culture with*[b] | *Anti-tumour effector cell alone* | *Tumour cell alone* | *E/T ratio*[c] | *Tumour + anti-tumour effector cells* *Expected*[d] | *Observed* |
| CTLs | 2,650 ± 132 | 1,367 ± 116 | 10:1 | 4,016 ± 29 | 3,083 ± 76 |
| LAK | 617 ± 29 | 1,550 ± 132 | 10:1 | 2,166 ± 153 | 25,000 ± 0[e] |
| NK | 1,150 ± 132 | 1,583 ± 76 | 10:1 | 2,717 ± 76 | 18,500 ± 866[e] |
| PMN | 4,100 ± 361 | 1,583 ± 76 | 200:1 | 5,683 ± 431 | 25,000 ± 0[e] |
| Activated macrophages | 4,150 ± 180 | 1,700 ± 100 | 10:1 | 5,850 ± 278 | 15,333 ± 1,155[e] |
| Resident macrophages | 1,767 ± 252 | 1,633 ± 153 | 50:1 | 3,400 ± 361 | 8,933 ± 603[e] |

[a]$PGE_2$ production was observed when 1 × $10^4$ QR-32 cells were co-cultured with effector cells at an E/T ratio which produced about 30% lysis of the QR-32 cells. $PGE_2$ levels in supernatants obtained from co-cultures in 24-well plastic plates for 48 h. [b]The methods for the induction and collection of each effector cell are described in Table I, footnotes c–h, and in the Materials and methods section. [c]Effector-to-target cell ratio. [d]Expected values were calculated from the additive production of $PGE_2$ by the tumour and effector cells. [e]Significant increase in the observed production of $PGE_2$ by the co-cultured cells was observed as compared with the expected values ($P < 0.001$).

effector cells ($P<0.001$). On the other hand, $PGE_2$ production by co-culture of tumour cells with CTLs is almost equal to the expected value. Not only QR-32 cells but also tumorigenic parental BMT-11 cl-9 cells, which produce large amounts of $PGE_2$ by themselves, can be converted so as to produce much greater amounts of $PGE_2$ after co-culture with anti-tumour effector cells (data not shown).

### *Inhibition of the increase in $PGE_2$ production during QR-32 cell co-culture with anti-tumour effector cells in the presence of radical scavengers*

We examined the effect of radical scavengers on the $PGE_2$ production of QR-32 cells enhanced by co-culturing them with anti-tumour effector cells (Figure 2). Superoxide dismutase (SOD, 300 U $ml^{-1}$) inhibited the increase in $PGE_2$ production after QR-32 cells were co-cultured with LAK and PMN cells ($P<0.001$ and $P<0.005$, respectively), whereas SOD did not inhibit $PGE_2$ production significantly after being co-cultured with NK cells or activated/resident macrophages. In the presence of catalase (20,000 U $ml^{-1}$) and mannitol (5 × $10^{-2}$ M), $PGE_2$ production was significantly inhibited when tumour cells were co-cultured with anti-tumour effector cells. As a positive control, $PGE_2$ production was also inhibited after we added indomethacin ($10^{-6}$ M), an inhibitor of prostaglandin synthesis.

**Figure 2** Inhibition of the increase in $PGE_2$ production during co-culture of QR-32 cells with anti-tumour effector cells in the presence of radical scavengers. Approximately 1 × $10^4$ QR-32 cells were co-cultured with various anti-tumour effector cells with or without the oxygen radical scavengers SOD (300 U $ml^{-1}$), catalase (20,000 U $ml^{-1}$) or mannitol (5 × $10^{-2}$ M) for 48 h. Each E/T ratio which produced about 30% lysis of QR-32 cells was determined. Determinations were carried out in triplicate and the mean and standard deviation were obtained. *$P<0.001$, **$P<0.005$; NS, not significant *vs* $PGE_2$ production by the corresponding no-scavenger group. ■, No scavengers; ▒, SOD; , ▨, catalase; ▧, mannitol, □, indomethacin; ▩, effector alone.

### *Effects of oxygen radical scavengers on the cytolysis of QR-32 cells during co-culture with lymphokine-activated killer cells*

Since LAK cells produce various species of oxygen radicals in our system (Figure 2), and since QR-32 cells have been shown to be highly sensitive to LAK cells even in a 6 h $^{51}Cr$-release assay (Okada *et al.*, 1990), we next examined the effects of oxygen radicals produced by LAK cells on the cytolysis of QR-32 cells (Table III). The LAK cells' killing activities were not significantly reduced during co-culture with SOD, catalase and mannitol. No cytotoxic activity by radical scavengers on QR-32 cells was observed.

### *Increased production of prostaglandin $E_2$ after exposure of QR-32 cells to IFN-α A/D, TNF-α and TGF-β*

We measured the $PGE_2$-producing activity and enhancement of cell growth of QR-32 cells after their co-culture with various recombinant cytokines and growth factors. IL-1β, IL-2, IL-6, G-CSF, IFN-α A/D, TNF-α, TGF-α and -β, bFGF and EGF were diluted to 10-fold dilutions from high concentrations and 1 × $10^4$ QR-32 cells were exposed to each dilution for 48 h, after which we measured the $PGE_2$ in the culture supernatants. Table IV shows typical data from one of at least two experiments. IFN-α A/D, TNF-α and TGF-β induced significantly increased $PGE_2$ production by the QR-32 cells ($P<0.001$). Cell growth was inhibited by as much as 59.8% and 61.0% when QR-32 cells were exposed to 100 and 10 ng $ml^{-1}$ IFN-α A/D respectively, by 54.8% and 77.7% when the cells were exposed to 1,000 and 100 U $ml^{-1}$ TNF-α respectively and by 88.7% when the cells were exposed to 10 ng $ml^{-1}$ TGF-β, all as compared with the growth of untreated QR-32 cells (100%). Other cytokines which did not induce QR-32 cells to produce large amounts of $PGE_2$ did not inhibit the growth of QR-32 cells either.

### Discussion

In this study, we have been able to demonstrate that prostaglandin $E_2$ ($PGE_2$) production by QR-32 cells is augmented when the tumour cells are co-cultured with various anti-tumour effector cells at various effector-to-tumour cell ratios, with the exception of cytotoxic T lymphocytes (CTLs) specific to the tumour cells. Enhanced $PGE_2$ production by

**Table III** Effects of oxygen radical scavengers on the cytolysis of QR-32 cells after co-culture with lymphokine-activated killer cells

| *Treated with*[a] | *Specific cytolysis of QR-32 cells after co-culture with (%)*[b] *LAK* | *Nothing* |
|---|---|---|
| Nothing | 68.9 ± 6.4 | – |
| SOD | 69.7 ± 5.6 | 0.0 ± 0.0 |
| Catalase | 66.7 ± 5.2 | 0.3 ± 0.3 |
| Mannitol | 64.7 ± 1.6 | 0.0 ± 0.0 |

[a]Each cell type was plated into wells in a 96-well plastic plate with or without oxygen radical scavengers; SOD (300 U $ml^{-1}$), catalase (20,000 U $ml^{-1}$) and mannitol ($5 \times 10^{-2}$ M) for 6 h. [b]The cytolytic activity was assessed in a 6 h $^{51}Cr$-release assay against QR-32 cells with an E/T ratio of 50:1. Determinations were carried out in triplicate.

**Table IV** $PGE_2$ production and growth of QR-32 cells after exposure to various cytokines and growth factors

| *QR-32 cells co-cultured with* | *Concentration ($ml^{-1}$)* | *$PGE_2$ production*[a] *(pg $ml^{-1}$)* | *Cell growth*[b] *(%)* |
|---|---|---|---|
| IFN-α A/D | 100 ng | 10,667 ± 1,155[c] | 59.8 |
| | 10 ng | 8,733 ± 231[c] | 61.0 |
| TNF-α | 1,000 U | All >25,000[c] | 54.8 |
| | 100 U | All >25,000[c] | 77.7 |
| TGF-β | 10 ng | 8,400 ± 173[c] | 88.7 |
| | 1 ng | 4,966 ± 252[c] | 101.7 |
| IL-1β | 100 ng | 2,633 ± 252[d] | 106.5 |
| | 10 ng | 2,617 ± 161[d] | 104.1 |
| IL-2 | 1,000 U | 2,550 ± 50[d] | 99.5 |
| | 100 U | 2,433 ± 58[d] | 100.0 |
| IL-6 | 50 U | 2,533 ± 116[d] | 95.0 |
| | 5 U | 2,517 ± 76[d] | 103.0 |
| G-CSF | 2,500 pg | 2,483 ± 76[d] | 102.5 |
| | 250 pg | 2,450 ± 87[d] | 101.7 |
| TGF-α | 10 ng | 2,833 ± 153[d] | 93.9 |
| | 1 ng | 2,483 ± 176[d] | 105.0 |
| bFGF | 500 ng | 2,533 ± 58[d] | 107.0 |
| | 100 ng | 2,517 ± 104[d] | 102.5 |
| EGF | 1,000 ng | 2,467 ± 58[d] | 102.4 |
| | 100 ng | 2,417 ± 104[d] | 100.0 |
| None | – | 2,550 ± 150 | 100.0 |
| Medium alone | – | All <250 | – |

[a]Approximately $1 \times 10^4$ QR-32 cells were plated into wells in a 24-well plastic plate with or without cytokine or growth factor. $PGE_2$ production in culture supernatant after 48 h was measured. [b]Per cent cell growth when compared with non-treated cell growth assessed by 48 h MTT assay. [c]$P < 0.001$. [d]Not significant *vs* $PGE_2$ production by QR-32 cells alone.

tumour cells is considered to be an important mechanism for facilitating tumour cell escape from host immune surveillance. We have previously reported that QR-32 cells derived from tumorigenic BMT-11 cl-9 cells, which produce large amounts of $PGE_2$, find it hard to grow progressively in normal syngeneic mice because of a decrease in the production of $PGE_2$ (Okada *et al.*, 1990). We have also observed that $PGE_2$ acts not only as an immunosuppressive factor but also as a positive factor for the chemotactic and motile behaviour of tumour cells (Young *et al.*, 1991). These previous observations revealed that enhanced $PGE_2$ production by tumour cells results in the malignant progression of the tumour cells (Okada *et al.*, 1992). QR-32 cells produce large amounts of $PGE_2$ when the tumour cells have been co-cultured with foreign body-reactive cells (Okada *et al.*, 1992). In this study, we therefore attempted to determine which cell type of the anti-tumour effector cells is involved in the induction of QR-32 cell progression.

At the present time, we have not established why QR-32 cells co-cultured with CTLs do not induce $PGE_2$ production under the same conditions as co-culturing with other effector cells. One possible explanation is that CTLs may completely kill all tumour cells that bind specifically to tumour antigen(s) (Triozzi, 1993; Yasumura *et al.*, 1993). This would mean that only those tumour cells which do not make contact with CTLs can survive. On the other hand, although the antigen non-specific anti-tumour effector cells are able to kill a large proportion of the QR-32 cells, they would also affect the surviving tumour cells. We speculate, therefore, that surviving QR-32 cells, after contact with antigen non-specific anti-tumour effector cells, are converted so as to produce large amounts of $PGE_2$. This speculation is supported by our finding that only cytotoxic cytokines (IFN-α, TNF-α and TGF-β) enhanced $PGE_2$ production by QR-32 cells (Table IV). Our preliminary data show that the surviving QR-32 cells, after *in vitro* co-culture with NK cells and LAK cells were converted to tumorigenic tumours in normal syngeneic mice after subcutaneous injection (data not shown). We believe that, under appropriate conditions, anti-tumour effector cells within tumour tissues might induce malignant progression of tumours through the enhanced production of $PGE_2$ in the microenvironment surrounding the tumour cells. Regardless of whether this is so or not, it is nonetheless an important finding that anti-tumour effector cells may convert benign tumour cells into more malignant ones.

We have previously reported that oxygen radicals are involved in the mechanisms responsible for $PGE_2$ production by tumour cells (Okada *et al.*, 1992). Results indicated that oxygen radicals might play a role in the *in vivo* malignant progression of QR-32 cells (Okada *et al.*, 1993). We observed in the present study that enhanced $PGE_2$ production by QR-32 cells after co-culture with anti-tumour effector cells was inhibited in the presence of radical scavengers. We found that oxygen radicals produced by host effector cells induce somatic mutations in QR-32 cells (Okada *et al.*, 1993). However, the oxygen radicals produced by LAK cells do not seem to be enough to kill QR-32 cells, as we found when we added radical scavengers extracellularly to the co-culture system. Therefore, we speculate that the quantity of oxygen radicals required to alter tumour properties is much smaller than the quantity necessary for direct tumour cell killing.

We have observed that QR-32 cells can also be altered to produce large amounts of $PGE_2$ when they are cultured with cytotoxic cytokines in the absence of anti-tumour effector cells. This finding is a strong indication that high levels of $PGE_2$ production are caused mainly by the tumour cells themselves. We wish to conclude that the factors which stimulate $PGE_2$ production by tumour cells are derived from anti-tumour effector cells and not from tumour cells themselves. Our findings lead us to suggest that an *in vitro* experimental system using QR-32 cells may be useful for the detection of tumour progression-enhancing factor(s).

This work was supported in part by a Grant-in Aid for Cancer Research from the Japanese Ministry of Health and Welfare. The authors would like to thank Drs Masanobu Kobayashi and Akio Nakane for various suggestions. We thank Dr Mark Micallef, Miss Masako Yanome and Mr Willie Jones for their valuable suggestions and kind English revision of this manuscript.

### References

ANDERSON, S.A., ISAKSON, P.C., PURE, E., MUIRHEAD, M., UHR, J.W. & VITETTA, E.S. (1981). Immunosuppression in a murine B cell leukemia (BCL1): role of an adherent cell in the suppression of primary *in vitro* antibody responses. *J. Immunol.*, **126**, 1603–1607.

BOSSLET, K., RUFFMANN, R., ALTEVOGT, P. & SCHIRRMACHER, V. (1981). A rapid method for the isolation of metastasizing tumour cells from internal organs with the help of isopycnic density-gradient centrifugation in percoll. *Br. J. Cancer*, **44**, 356–362.

CATALONA, W.J. & CHRETIEN, P.B. (1973). Abnormalities of quantitative dinitrochlorobenzene sensitization in cancer patients: correlation with tumor stage and histology. *Cancer*, **31**, 353–356.

HOJO, H. & HASHIMOTO, Y. (1981). Cytotoxic cells induced in tumor-bearing rats by a streptococcus preparation (OK-432). *Gann*, **72**, 692–699.

ISHIKAWA, M., OKADA, F., HAMADA, J.-I., HOSOKAWA, M. & KOBAYASHI, H. (1987*a*). Changes in the tumorigenic and metastatic properties of tumor cells treated with quercetin or 5-azacytidine. *Int. J. Cancer*, **39**, 338–342.

ISHIKAWA, M., HOSOKAWA, M., OH-HARA, N., NIHO, Y. & KOBAYASHI, H. (1987*b*). Marked granulocytosis in C57BL/6 mice bearing a transplanted BMT-11 fibrosarcoma. *J. Natl Cancer Inst.*, **78**, 567–571.

JESSUP, J.M., COHEN, M.H., TOMASZEWSKI, M.M. & FELIX, E.L. (1976). Effects of murine tumors on delayed hypersensitivity to dinitrochlorobenzene. I. Description of anergy caused by transplanted tumors. *J. Natl Cancer Inst.*, **57**, 1077–1084.

KAWAGUCHI, T., SUEMATSU, M., KOIZUMI, H.M., MITSUI, H., SUZUKI, S., MATSUNO, T., OGAWA, H. & NOMOTO, K. (1983). Activation of macrophage function by intraperitoneal administration of the streptococcal antitumor agent OK-432. *Immunopharmacology*, **6**, 177–189.

KAWATA, A., HOSOKAWA, M., SAWAMURA, Y., ITO, K., UNE, Y., SHIBATA, T., UCHINO, J. & KOBAYASHI, H. (1990). Modification of lymphokine-activated killer cell accumulation into tumor sites by chemotherapy, local irradiation, or splenectomy. *Mol. Biother.*, **2**, 221–227.

LYNCH, N.R. & SALOMON, J.-C. (1979). Tumor growth inhibition and potentiation of immunotherapy by indomethacin in mice. *J. Natl Cancer Inst.*, **62**, 117–121.

MIZOBE, F., MARTIAL, E., COLBY-GERMINARIO, S. & LIVETT, B.G. (1982). An improved technique for the isolation of lymphocytes from small volumes of peripheral mouse blood. *J. Immunol. Methods*, **48**, 269–279.

MOSMANN, T. (1983). Rapid colorimetric assay for cellular growth and survival: application to proliferation and cytotoxicity assays. *J. Immunol. Methods*, **65**, 55–63.

OKADA, F., HOSOKAWA, M., HASEGAWA, J., ISHIKAWA, M., CHIBA, I., NAKAMURA, Y. & KOBAYASHI, H. (1990). Regression mechanisms of mouse fibrosarcoma cells after *in vitro* exposure to quercetin: diminution of tumorigenicity with a corresponding decrease in the production of prostaglandin $E_2$. *Cancer Immunol. Immunother.*, **31**, 358–364.

OKADA, F., HOSOKAWA, M., HAMADA, J.-I., HASEGAWA, J., KATO, M., MIZUTANI, M., REN, J., TAKEICHI, N. & KOBAYASHI, H. (1992). Malignant progression of a mouse fibrosarcoma by host cells reactive to a foreign body (gelatin sponge). *Br. J. Cancer*, **66**, 635–639.

OKADA, F., KOBAYASHI, H., HAMADA, J., TAKEICHI, N. & HOSOKAWA, M. (1993). Active radicals produced by host reactive cells in the malignant progression of murine tumor cells. *Proc. Am. Assoc. Cancer Res.*, **34**, 1060.

ROLLAND, P.H., MARTIN, P.M., JACQUEMIER, J., ROLLAND, A.M. & TOGA, M. (1980). Prostaglandin in human breast cancer: evidence suggesting that an elevated prostaglandin production is a marker of high metastatic potential for neoplastic cells. *J. Natl Cancer Inst.*, **64**, 1061–1070.

TRIOZZI, P.L. (1993). Identification and activation of tumor-reactive cells for adoptive immunotherapy. *Stem Cells*, **11**, 204–211.

WALKER, C., KRISTENSEN, F., BETTENS, F. & DEWECK, A.L. (1983). Lymphokine regulation of activated (GI) lymphocytes. I. Prostaglandin $E_2$-induced inhibition of interleukin 2 production. *J. Immunol.*, **130**, 1770–1773.

YASUMURA, S., HIRABAYASHI, H., SCHWARTZ, D.R., TOSO, J.F., JOHNSON, J.T., HERBERMAN, R.B. & WHITESIDE, T.L. (1993). Human cytotoxic T-cell lines with restricted specificity for squamous cell carcinoma of the head and neck. *Cancer Res.*, **53**, 1461–1468.

YOUNG, M.R. & DIZER, M. (1983). Enhancement of immune function and tumor growth inhibition by antibodies against prostaglandin $E_2$. *Immunol. Commun.*, **12**, 11–23.

YOUNG, M.R. & KNIES, S. (1984). Prostaglandin E production by Lewis lung carcinoma: mechanism for tumor establishment *in vivo*. *J. Natl Cancer Inst.*, **72**, 919–922.

YOUNG, M.R.I., OKADA, F., TADA, M., HOSOKAWA, M. & KOBOYASHI, H. (1991). Association of increased tumor cell responsiveness to prostaglandin $E_2$ with more aggressive tumor behavior. *Invasion Metastasis*, **11**, 48–57.